# Two new species of *Oobius* Trjapitzin (Hymenoptera, Encyrtidae) egg parasitoids of *Agrilus* spp. (Coleoptera, Buprestidae) from the USA, including a key and taxonomic notes on other congeneric Nearctic taxa

**DOI:** 10.3897/zookeys.498.9357

**Published:** 2015-04-21

**Authors:** Serguei V. Triapitsyn, Toby R. Petrice, Michael W. Gates, Leah S. Bauer

**Affiliations:** 1 Entomology Research Museum, Department of Entomology, University of California, Riverside, California, 92521, USA; 2 United States Department of Agriculture Forest Service, Northern Research Station, 3101 Technology Blvd., Suite F, Lansing, Michigan, 48910, USA; 3 Systematic Entomology Laboratory c/o National Museum of Natural History, P.O. Box 37012, Washington, DC, 20013-7012, USA

**Keywords:** Emerald ash borer, new species, congener identification key, *Oobius agrili*, Nearctic, egg parasitoid, biological control

## Abstract

*Oobius* Trjapitzin (Hymenoptera, Encyrtidae) species are egg parasitoids that are important for the biological control of some Buprestidae and Cerambycidae (Coleoptera). Two species, *Oobius agrili* Zhang & Huang and *Oobius longoi* (Siscaro), were introduced into North America for classical biocontrol and have successfully established. Two new native North American species that parasitize eggs of *Agrilus* spp. (Buprestidae) are described and illustrated from the USA: *Oobius minusculus* Triapitsyn & Petrice, **sp. n.** (Michigan), an egg parasitoid of both *Agrilus subcinctus* Gory on ash (*Fraxinus* spp.) and *Agrilus egenus* Gory on black locust (*Robinia pseudoacacia* L.) trees, and *Oobius whiteorum* Triapitsyn, **sp. n.** (Pennsylvania), an egg parasitoid of *Agrilus anxius* Gory on European white birch (*Betula pendula* Roth). A taxonomic key and notes on the Nearctic native and introduced *Oobius* species are also included.

## Introduction

The rather poorly known encyrtid genus *Oobius* Trjapitzin (Hymenoptera: Encyrtidae) currently includes 41 species worldwide, and seven are known from North America (Noyes 2014). Noyes (2010) recently described 20 of these species from Costa Rica and compared some of these new species to similar Nearctic taxa. Also, in Noyes (2010) the genera *Avetianella* Trjapitzin, *Szelenyiola* Trjapitzin, and *Oophagus* Liao were synonymized under *Oobius*.

As egg parasitoids of Buprestidae, Cerambycidae (Coleoptera; Noyes 2014) and Asilidae (Diptera; Annecke 1967), species of *Oobius* are important for the natural and classical biological control of some coleopteran species. Two species are being used as biological control agents in the USA where they are successfully established: *Oobius agrili* Zhang & Huang and *Oobius longoi* (Siscaro). The former was collected from China and was first released into the USA in 2007 as a biocontrol agent of the emerald ash borer, *Agrilus planipennis* Fairmaire (Buprestidae) (Bauer et al. in press). *Agrilus planipennis* is an invasive pest from Asia that attacks ash trees (*Fraxinus* spp.) (Haack et al. 2002; Bray et al. 2011). Releases of *Oobius agrili* are ongoing throughout infested regions of the USA. As of March 2015, establishment of *Oobius agrili* has been confirmed in Indiana, Maryland, Michigan, New York, Ohio, and Pennsylvania (Abell et al. 2014; Bauer et al. in press). *Oobius longoi* was introduced from Australia to California, USA, as a biocontrol agent for management of *Phoracantha recurva* Newman and *Phoracantha semipunctata* (Fabricius) (Cerambycidae), which are invasive pests of *Eucalyptus* trees in the USA (Hanks et al. 1995; Luhring et al. 2000).

Here two new species of *Oobius* are reported and a taxonomic key to the known native and introduced species of *Oobius* in North America is provided. One of the newly described species was reared initially from eggs of the native buprestid *Agrilus subcinctus* Gory in Michigan, whose larvae feed on the dead twigs of ash trees. This parasitoid was previously reported by Petrice et al. (2009) as *Avetianella* sp. Later, the second author of the current paper also reared this same species from eggs of *Agrilus egenus* Gory on black locust trees (*Robinia pseudoacacia* L.) in Michigan. *Agrilus egenus* is a native species that oviposits on dead or dying branches of black locust (Nelson et al. 2008; MacRae 1991). The second newly described *Oobius* species was reared from *Agrilus anxius* Gory eggs on European white birch (*Betula pendula* Roth) in Pennsylvania, based on the voucher specimens from the study by Loerch and Cameron (1983). *Agrilus anxius* is a native species that attacks both native and introduced birch trees (*Betula* spp.) in North America (Nelson et al. 2008).

## Material and methods

**Collecting and rearing new species of *Oobius*.** Ash tree twigs with *Agrilus subcinctus* eggs and black locust twigs with *Agrilus egenus* eggs were collected in the field in Ingham and Clinton counties, Michigan in 2013 and 2014. Eggs were monitored in the laboratory for parasitoid emergence. Voucher specimens of the parasitoids were preserved in 95% ethanol and sent to the senior author for identification. See Loerch and Cameron (1983) for collection of parasitoids from *Agrilus anxius*.

**Taxonomic studies.** Parasitoid specimens used in the taxonomic studies were critical point dried from ethanol and point-mounted. Selected specimens were then dissected and slide-mounted in Canada balsam, examined under a Zeiss®™ Axioskop 2 plus compound microscope using Nomarski differential interference contrast optics. Stereomicroscopic images were compiled with Auto-Montage 4.02 (Synchroscopy®™) to illustrate select specimens. Images of specimens were produced by scanning electron microscopy (SEM) and an EntoVision Imaging Suite. A Nikon®™ SMZ1500 and Leica®™ MZ 9.5 stereomicroscope with 10X oculars (Nikon C-W10X/22) and Chiu Technical Corp.®™ Lumina 1 FO-150 and fiber optic light source was used for pinned specimen observation. Mylar film was placed over the ends of the light source to reduce glare. Scanning electron microscope (SEM) images were taken with a Hitachi®™ TM3000 desktop unit (Tungsten source). Some specimens were manually cleaned of external debris with forceps or brushes and affixed to 12.7X 3.2 mm Leica/Cambridge aluminum SEM stubs with carbon adhesive tabs (Electron Microscopy Sciences, #77825-12). Stub-mounted specimens were imaged uncoated or sputter coated using a Cressington Scientific 108 Auto with a gold-palladium mixture from at least three different angles to ensure complete coverage (~20–30nm coating). Color images were obtained using an EntoVision Imaging Suite, which includes a firewire JVC KY-75 3CCD digital camera mounted to a Leica M16 zoom lens via a Leica z-step microscope stand. Slides of *Oobius buprestidis* and *Oobius dahlsteni* were imaged with a Leica DMRB compound microscope fitted with Leica HCX PL “Fluotar” 5× and 10× metallurgical grade lenses. Both systems fed image data to a desktop computer where Cartograph 5.6.0 (Microvision Instruments®™, France) was used to capture a fixed number of focal planes (based on magnification); the resulting focal planes (manually captured via Archimed 5.5.0 on the DMRB) were merged into a single, in-focus composite image. Uniform lighting was achieved using a LED illumination dome with all four quadrants set to 99.6% intensity. The images were then retouched where necessary using Adobe Photoshop®™ CS4/CS6 with plates assembled using InDesign CS4/CS6.

Terms used for morphological features are those of Gibson (1997). Abbreviations used are: F = antennal funicle segment; mps = multiporous plate sensillum or sensilla on the antennal flagellar segments (= longitudinal sensillum or sensilla or sensory ridge(s) of authors). Body length was measured without the exserted part of the ovipositor.

Acronyms for depositories of specimens are as follows: BMNH, The Natural History Museum, London, England, UK; EMEC, Essig Museum of Entomology, University of California, Berkeley, California, USA; IZCAS, Institute of Zoology, Chinese Academy of Sciences, Beijing, China; MSUC, Albert J. Cook Arthropod Research Collection, Department of Entomology, Michigan State University, East Lansing, Michigan, USA; PSUC, Frost Entomological Museum, Pennsylvania State University, University Park, State College, Pennsylvania, USA; UANL, Universidad Autónoma de Nuevo León, San Nicolás de los Garza, Monterrey, Mexico; UCRC, Entomology Research Museum, University of California, Riverside, California, USA; UNCA, Institute of Agricultural Entomology, University of Catania, Catania, Sicily, Italy; USNM, National Museum of Natural History, Washington, District of Columbia, USA.

## Taxonomy

### Oobius

Taxon classificationAnimaliaHymenopteraEncyrtidae

Trjapitzin, 1963

Oobius Trjapitzin 1963: 544–545. Type species: *Tyndarichus rudnevi* Nowicki, by original designation.
Avetianella Trjapitzin 1968: 97–99. Type species: *Avetianella capnodiobia* Trjapitzin, by monotypy. Synonymized under *Oobius* by Noyes 2010: 660–662.
Szelenyiola Trjapitzin 1977: 160. Type species: *Szelenyiola nearctica* Trjapitzin, by original designation and monotypy. Synonymized under *Oobius* by Noyes 2010: 660–662.
Oobius : Trjapitzin 1977: 161 (key to genera of the subtribe Oobiina of the tribe Discodini of the subfamily Encyrtinae); Noyes 2010: 660–662 (synonymy, diagnosis, host associations, comments); Trjapitzin and Volkovitsh 2011: 670–672 (diagnosis of *Oobius* s. str., taxonomic position, key to world species).
Avetianella : Gordh and Trjapitzin 1981: 6 (comments); Trjapitzin 2001: 734–735 (comments, key to world species).
Oophagus Liao in Liao et al. 1987: 184. Type species: *Oophagus batocerae* Liao, by original designation and monotypy. Synonymized under *Avetianella* by Zhang and Huang 2004: 34–35, and under *Oobius* by Noyes 2010: 660.
Szelepyiola : Trjapitzin and Volkovitsh 2011: 671 (misspelled).

#### Comments.

*Oobius* is a cosmopolitan genus as defined by Noyes (2010) who provided its detailed diagnosis, which is omitted here for brevity. One extralimital species, *Oobius striatus* Annecke, is also known from eggs of Asilidae (Diptera) in Montenegro and Zimbabwe (Annecke 1967; Noyes 2010, 2014).

#### Key to the Nearctic species of *Oobius*, females (both native and introduced)

(*Oobius depressus* (Girault) not included)

**Table:** 

1	Tarsi 4-segmented (Fig. 1)	***Oobius agrili* Zhang & Huang**
–	Tarsi 5-segmented (Figs 7, 13)	**2**
2(1)	Clava entire (Figs 9, 10)	***Oobius nearcticus* (Trjapitzin)**
–	Clava 3-segmented (Figs 2, 6, 8, 12, 22)	**3**
3(2)	Body length (dry-mounted specimens) at most 0.53 mm; mps only on F6 (Fig. 12)	***Oobius minusculus* sp. n.**
–	Body length (dry-mounted specimens) at least 0.66 mm; mps on F6 and other funicle segments (Figs 6, 20, 22)	**4**
4(3)	Mps on F5 and F6 (Fig. 6)	***Oobius buprestidis* (Gordh & Trjapitzin)**
–	Mps on F4–F6 (Figs 8, 22)	**5**
5(4)	Linea calva “open” posteriorly (Fig. 23), uninterrupted by row of setae	***Oobius longoi* (Siscaro)**
–	Linea calva interrupted posteriorly by a line (or lines) of setae (Figs 19, 27)	**6**
6(5)	F5 and F6 each notably longer than F4 (Fig. 26), F4 0.8× length of F5	***Oobius whiteorum* sp. n.**
–	F5 and F6 each subequal in length to F4 (Fig. 8), F4 more than 0.9× length of F5	***Oobius dahlsteni* (Trjapitzin)**

**Figures 1–8.**
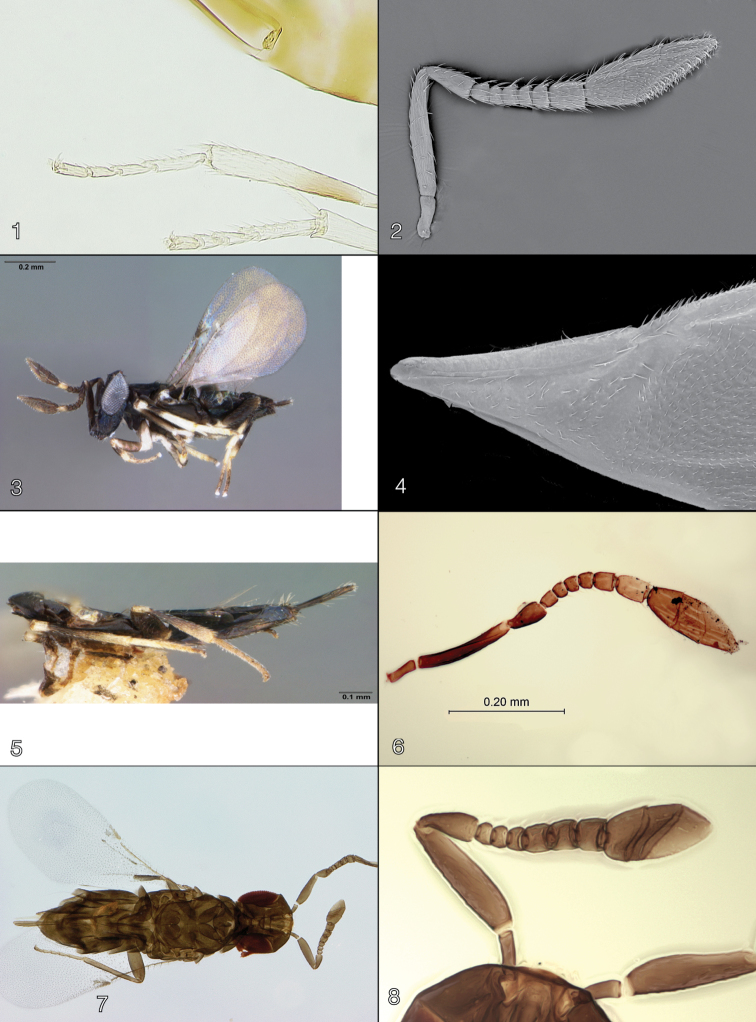
**1–4** *Oobius agrili* female (from USDA Forest Service laboratory colony, East Lansing, Michigan, USA; of China origin), **1** hind leg **2** antenna **3** lateral habitus **4** forewing base **5–6** *Oobius buprestidis* female (holotype), **5** lateral habitus **6** antenna **7–8** *Oobius dahlsteni* female (holotype) **7** dorsal habitus **8** antenna.

**Figures 9–16.**
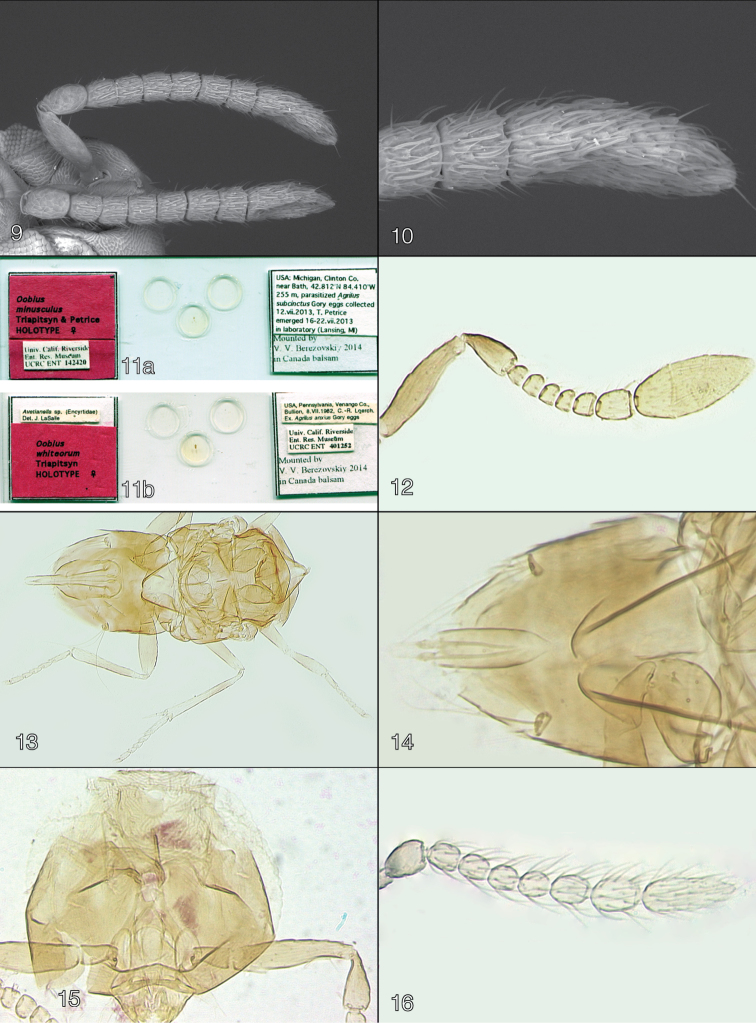
**9–10** *Oobius nearcticus* female (holotype), **9** antenna **10** clava **11a–11b: 11a** holotype slide of *Oobius minusculus* **11b** holotype slide of *Oobius whiteorum* **12–16** *Oobius minusculus* **12** antenna (holotype female) **13** mesosoma and metasoma (holotype female) **14** metasoma (paratype male) **15** head (paratype female) **16** pedicel and flagellum (paratype male).

**Figures 17–24.**
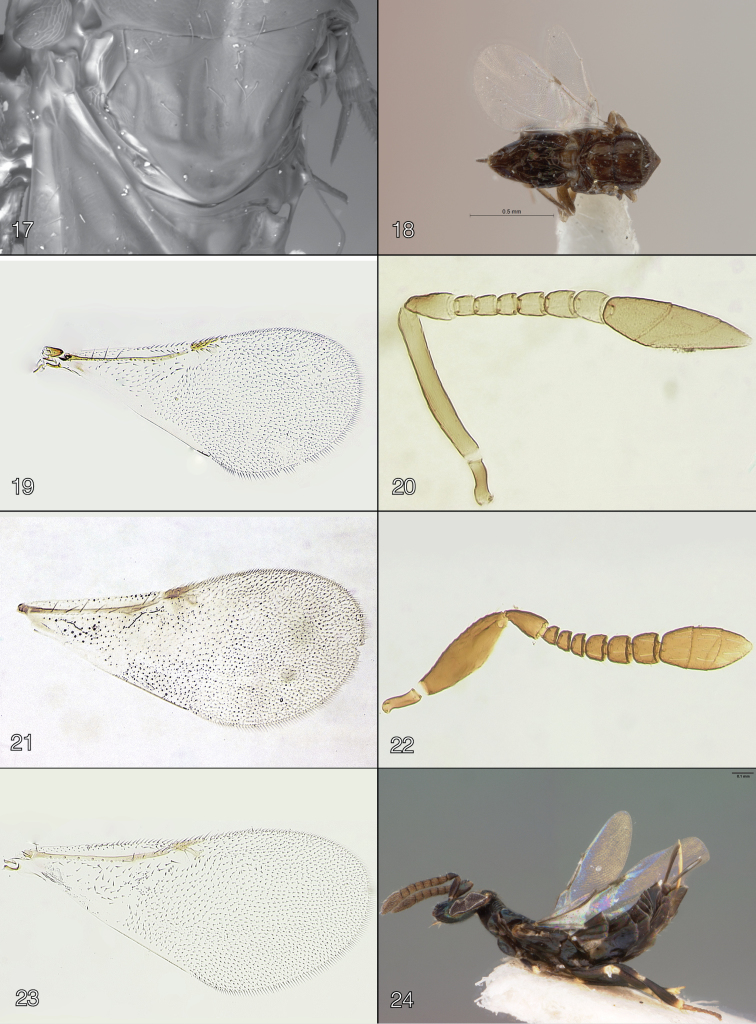
**17–18** *Oobius depressus* female **17** dorsal scutellum (lectotype) **18** dorsal habitus (paralectotype) **19** *Oobius zahaikevitshi* female (environs of Volgograd, Krasnoarmeyskiy District, Volgograd Province, Russia), forewing **20–21** *Oobius hasmik* female (paratype) **20** antenna **21** forewing **22–23** *Oobius longoi* female (from University of California laboratory colony, Riverside, California, USA; of Australia origin), **22** antenna **23** forewing **24** *Oobius nearcticus* female (holotype), lateral habitus.

**Figures 25–32.**
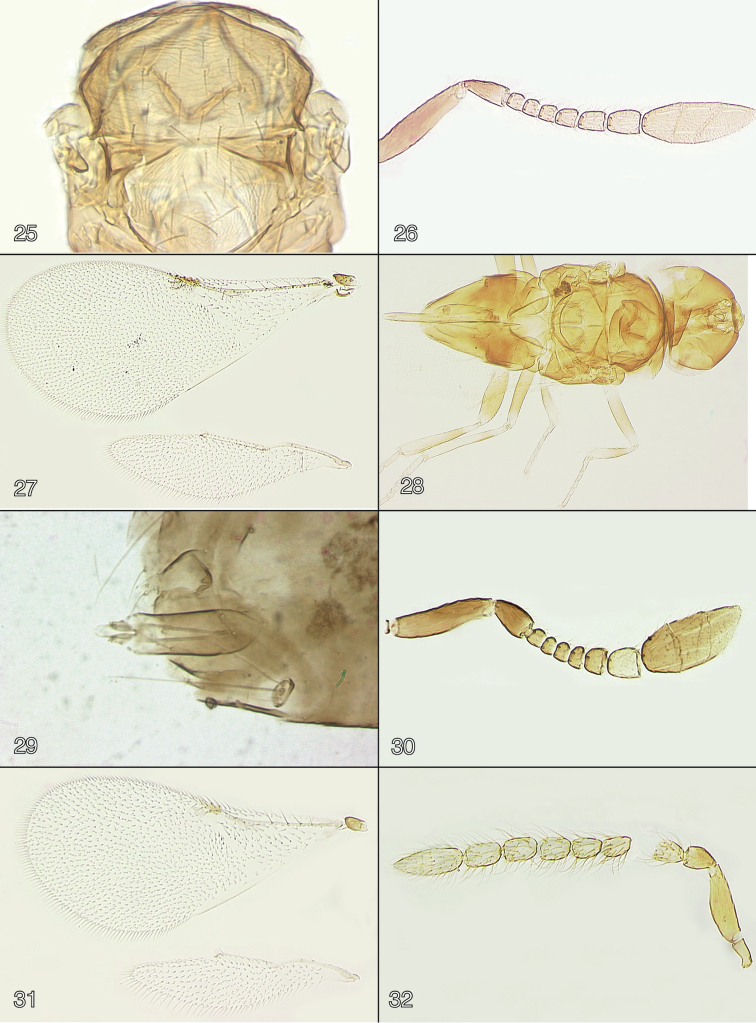
**25** *Oobius minusculus* male (paratype), mesosoma **26–29** *Oobius whiteorum* **26** antenna (holotype female) **27** a pair of wings (holotype female) **28** dorsal habitus (holotype female) **29** genitalia (paratype male) **30** *Oobius zahaikevitshi* female (environs of Volgograd, Krasnoarmeyskiy District, Volgograd Province, Russia), antenna **31** *Oobius minusculus* female (holotype), a pair of wings **32** *Oobius whiteorum* (paratype male), antenna.

### Alphabetical synopsis of the Nearctic species

#### Oobius agrili

Taxon classificationAnimaliaHymenopteraEncyrtidae

Zhang & Huang, 2005

Figures 1–4

Oobius agrili Zhang & Huang in Zhang et al. 2005: 254–258. Holotype female [IZCAS], not examined. Type locality: Changchun, Jilin, China.
Oobius agrili Zhang & Huang: Trjapitzin and Volkovitsh 2011: 671 (key), 672–673 (taxonomic history, host associations, use in biological control for *Agrilus planipennis*).

##### Material examined.

USA, Michigan, Ingham Co., East Lansing, United States Department of Agriculture (USDA) Forest Service Northern Research Station, laboratory culture of *Oobius agrili* reared in *Agrilus planipennis* eggs: 37^th^-generation progeny, emerged 10.viii.2014, D.L. Miller, originally from CHINA, Jilin (Jingyuetan Forest Park, Changchun), 2006, T. Zhao (Zhao Tonghai), from eggs of *Agrilus planipennis* [10 ♀, UCRC]; 6–7^th^-generation progeny, emerged 31.vii.2014, D.L. Miller, originally from CHINA, Jilin (Jingyuetan Forest Park, Changchun), 2008, T. Zhao, from eggs of *Agrilus planipennis* [11 ♀, UCRC]; 4–8^th^-generation progeny, emerged 10.viii.2014, D.L. Miller, originally from CHINA, Jilin (Jingyuetan Forest Park, Changchun), 2009, T. Zhao, from eggs of *Agrilus planipennis* [16 ♀, UCRC]; 9^th^-generation progeny, emerged 18.vii.2014, D.L. Miller, originally from CHINA, Jilin (Jingyuetan Forest Park, Changchun), 2008, T. Zhao, from eggs of *Agrilus planipennis* [11 ♀, UCRC].

##### Distribution.

China (Zhang et al. 2005; Liu et al. 2007); USA (introduced): Indiana, Maryland, Michigan, New York, Ohio, and Pennsylvania, as of March 2015 (Abell et al. 2014; Bauer et al. in press).

##### Host.

*Agrilus planipennis* Fairmaire.

##### Comments.

*Oobius agrili* is a solitary thelytokous egg parasitoid of *Agrilus planipennis*, discovered in 2004 during foreign exploration for natural enemies in northeast China (Zhang et al. 2005; Liu et al. 2007; Trjapitzin and Volkovitsh 2011). Adults *Oobius agrili* were reared from eggs at the USDA Forest Service Northern Research Station laboratory in East Lansing, Michigan, USA. Rearing stock for this colony originated from parasitized *Agrilus planipennis* eggs collected from *Fraxinus pennsylvanica* trees in Jingyuetan Forest Park, Changchun, Jilin Province, China in 2004–2009. In 2007, *Oobius agrili* introductions began in Michigan, USA, for classical biological control of *Agrilus planipennis*. As of fall 2014, releases of *Oobius agrili* had expanded to 19 states (Bauer et al. in press). Abell et al. (2014) reported parasitism of *Agrilus planipennis* eggs averaged approximately 20% in 2012–2013 at some sites where *Oobius agrili* was established, however, more studies are needed to assess the impact of *Oobius agrili* and other *Agrilus planipennis* biocontrol agents on ash recovery in the USA. Since 2010, stock cultures of *Oobius agrili* have been provided to the USDA Animal and Plant Health Inspection Service Emerald Ash Borer Biocontrol Facility, Brighton, Michigan, USA, for mass-rearing and releasing as a biocontrol agent of *Agrilus planipennis* in infested regions of the USA (Mapbiocontrol 2014). To distinguish *Oobius agrili* from the known native and the other introduced *Oobius* species, we provide illustrations of its metatarsus (Fig. 1), female antenna (Fig. 2), lateral habitus of the female (Fig. 3), and base of the forewing (Fig. 4).

#### Oobius buprestidis

Taxon classificationAnimaliaHymenopteraEncyrtidae

(Gordh & Trjapitzin, 1981)

Figures 5–6

Avetianella buprestidis Gordh and Trjapitzin 1981: 7–8, 9 (key), 59 (illustrations). Type locality: Portland, Multnomah Co., Oregon, USA.
Avetianella buprestidis : Trjapitzin 2001: 735 (key), 736 (list).
Oobius buprestidis (Gordh & Trjapitzin): Noyes 2010: 662.

##### Type material examined.

Holotype female [USNM] on point mount labeled with following seven labels: “Ex egg of Bupretus [sic] aurulentus”, “Portland, Ore., F.D. Keen Colr.”, “Hopk. US No. 33150-D”, “Lot No. 41-14524”, “*Habrolepoidea* n. sp. det. Gahan”, “*Avetianella* sp.n. Det Trjapitzin et Gordh”, [red] “Holotypus *Avetianella buprestidis* G. & T.”. The head and antenna are slide mounted separately: [left label] “♀ Holotype, Head & antenna, *Avetianella buprestidis* Gordh & Trjapitzin”, [right label] “Portland, Oregon, Hopkins #33150-D, Lot #41-14524, Ex eggs *Buprestus aurulentus*”. The forewing is mounted on an additional slide with the forewing of a male paratype: [left label] “♂ Forewing, top, *Avetianella buprestidis* G.&T., Portland, Ore., Lot # 41-14524, Hopkins # 33150-D, ♂ paratype”, [right label] “♀ Forewing, bottom, (Holotype) Ex. eggs *Buprestus aurulentus*, F.P. Keen, col. Head & antenna, *Avetianella buprestidis* Gordh & Trjapitzin, [right label] “Portland, Oregon, Hopkins #33150-D, Lot #41-14524, Ex eggs *Buprestus aurulentus*”.

##### Distribution.

USA (Oregon) (Gordh and Trjapitzin 1981).

##### Host.

*Buprestis aurulenta* L. (Gordh and Trjapitzin 1981 [as *Buprestus aurulentus*]; Trjapitzin 2001 [as *Cypriacus aurilentus* L.]).

##### Comments.

The point-mounted portion of the type (Fig. 5) is positioned at the apex of the point. Co-mounted proximally is a complete male paratype. A sliver of wood is pinned in the main collection (USNM) on which are eight eggs of *Buprestis aurulenta* (7 of which have parasitoids emergence holes) bearing the 33150-D Hopkins number designation.

#### Oobius dahlsteni

Taxon classificationAnimaliaHymenopteraEncyrtidae

(Trjapitzin, 1971)

Figures 7–8

Avetianella dahlsteni Trjapitzin 1971: 890–892. Type locality: McCloud [Flat], Siskiyou Co., California, USA.
Avetianella dahlsteni : Gordh and Trjapitzin 1981: 9 (key); Trjapitzin and Gordh 1984: 1275; Trjapitzin 2001: 735 (key), 736 (list).
Oobius dahlsteni (Trjapitzin): Noyes 2010: 662.

##### Type material examined.

Holotype female [EMEC] on slide labeled: [left label] “*Avetianella dahlsteni* Trjapitzin ♀, Trjapitzin 1970, CFL III-69, Ch. phenol gum damar, Div. Biol. Conn. Univ. Calif [“holotype” handwritten at top, middle, and bottom of label in red ink]”, [right label] “McCloud Flat, Siskiyou Co. Calif., July, 1968, *Agrilus brevicornis* rearing carton, A900, MF2-5 SR., D. L. Dahlsten”.

##### Distribution.

USA (California) (Trjapitzin 1971).

##### Hosts.

Unknown.

##### Comments.

The holotype (Fig. 7) is complete and whole mounted; its antenna (Fig. 8) is also illustrated to facilitate recognition of this species.

#### Oobius depressus

Taxon classificationAnimaliaHymenopteraEncyrtidae

(Girault, 1916)

Figures 17–18

Habrolepoidea depressa Girault 1916: 343–344. Type locality: Morristown, Henry Co., Illinois, USA.
Avetianella depressa (Girault): Gordh and Trjapitzin 1981: 8; Trjapitzin and Gordh 1984: 1275 (lectotype designation, comments); Trjapitzin 2001: 735 (mentioned), 736 (list). *Oobius depressus* (Girault): Noyes 2010: 662, 690.

##### Type material examined.

Lectotype female [USNM], designated by Trjapitzin & Gordh (1984), on point with following six labels: “Morristown XII-8-14 Ill”, “ExEggs Cylene robinae”, “JRMalloch Coll.”, [red] “Paratype No. 20328 U. S. N. M.”, “*Avetianella* Det. Trjapitzin et Gordh”, [red] “*Lectotypus* ♀ *Habrolepoidea depressa* Grlt Des. Trjapitzin et Gordh”. Paralectotypes, 2 males, 2 females: 1 female [USNM] on point with following six labels: “Morristown XII-8-14 Ill”, “ExEggs Cylene robinae”, “JRMalloch Coll.”, [red] “Paratype No. 20328 U. S. N. M.”, [red] “*Paralectotypus* ♀ *Habrolepoidea depressa* Grlt Des. Trjapitzin et Gordh”, “*Avetianella depressa* (Girault) ♀ Det. V. Trjapitzin May 1997”; 1 female [USNM] on point with following six labels: “Morristown XII-8-14 Ill”, “ExEggs *Cylene robinae*”, “JRMalloch Coll.”, [red] “Paratype No. 20328 U. S. N. M.”, 5. “*Habrolepoidea depressa* Gir Type”, “LECTOTYPE *Habrolepoidea depressa* Girault By B.D. Burks”; 2 males [USNM] on points, each with following four labels: “Morristown XII-8-14 Ill”, “ExEggs *Cylene robinae*”, “JRMalloch Coll.”, [red] “Paratype No. 20328 U. S. N. M.”. All specimens of the type series lack the heads and antennae (Trjapitzin and Gordh 1984; Trjapitzin 2001).

##### Distribution.

USA (Illinois) (Girault 1916).

##### Host.

*Megacyllene robiniae* (Forster) (Cerambycidae) (Girault 1916 [as *Cyllene robiniæ*]).

##### Comments.

The identity of this species remains unclear because the original description is poor and without any illustrations; unfortunately, the slide with a head and a forewing of each sex (Girault 1916) could not be found in the USNM and is presumed lost. The lectotype label affixed by B. D. Burks was not validly designated and is merely a paralectotype. To facilitate identification of this species, we provide illustrations of its scutellum (Fig. 17) and habitus of the female in dorsal view (Fig. 18).

#### Oobius longoi

Taxon classificationAnimaliaHymenopteraEncyrtidae

(Siscaro, 1992)

Figures 22–23

Avetianella longoi Siscaro 1992: 206–211. Holotype female [UNCA], not examined. Type locality: Grammichele, Catania Prov., Sicily, Italy.
Avetianella longoi : Trjapitzin 2001: 735 (key), 737–738 (taxonomic history, host associations); Wang et al. 2008: 1772–1777 (host associations, morphological and molecular data).
Oobius longoi (Siscaro): Noyes 2010: 662, 692.

##### Material examined.

Australia, New South Wales, Corowa, 22.i.2006, Q. Wang, from eggs of *Phoracantha recurva* [1 ♀, 1 ♂, UCRC]. Portugal: Lisboa, Montijo, Pegões, viii.1992, P. Albino, M. R. Paiva, from eggs of *Phoracantha semipunctata* [9 ♀, 11 ♂, UCRC]. Viseu, Villa Cova à Coelheira, viii.1992, P. Albino, M. R. Paiva, from eggs of *Phoracantha semipunctata* [11 ♀, UCRC]. USA, California, Riverside Co., Riverside, University of California campus, Department of Entomology Insectary, laboratory culture on eggs of *Phoracantha semipunctata* on *Eucalyptus* sp.: 29.ix.1994, L. Hanks (originally from Australia) [4 ♀, UCRC]; 1998, S. McElfresh, J. Gould (originally from: Australia, Victoria, Melbourne, Bundoora, La Trobe Wildlife Sanctuary, i.1992, Q. Wang, from eggs of *Phoracantha semipunctata* on fallen *Eucalyptus* sp.) [25 ♀, 22 ♂, UCRC].

##### Distribution.

Australia (indigenous); introduced (in some cases possibly unintentionally) into Hungary, Italy, Portugal, South Africa, Spain, USA (California), and Zambia (Trjapitzin 2001; Noyes 2014).

##### Hosts.

*Phoracantha recurva* Newman and *Phoracantha semipunctata* (Fabricius) (Cerambycidae) in California, USA (Wang et al. 2008); its other longhorned beetle hosts in Australia are listed by Trjapitzin (2001) and Noyes (2014).

##### Comments.

*Oobius longoi* is well known as an effective biological control agent and a successfully established parasitoid of *Phoracantha recurva* and *Phoracantha semipunctata* in California and elsewhere in the world (Hanks et al. 1995; Luhring et al. 2000; Trjapitzin 2001).

#### Oobius minusculus

Taxon classificationAnimaliaHymenopteraEncyrtidae

Triapitsyn & Petrice sp. n.

http://zoobank.org/A7698FE3-D6BF-4AB1-B796-9D006B040D45

Figures 11a , 12–16 , 25 , 31 , 33–34

Avetianella sp.: Petrice et al. 2009: 179–180 (egg parasitoid of *Agrilus subcinctus* in Livingston Co., Michigan, USA).

##### Type material.

Holotype female [UCRC] on slide (Fig. 11a) with following four labels: “USA: Michigan, Clinton Co., near Bath, 42.812°N, 84.410°W, 255 m, parasitized *Agrilus subcinctus* Gory eggs collected 12.vii.2013, T.R. Petrice, emerged 16-22.vii.2013 in laboratory (Lansing, MI)”, “Mounted by V. V. Berezovskiy 2014 in Canada balsam”, [magenta] “*Oobius minusculus* Triapitsyn & Petrice HOLOTYPE ♀”, [database label] “Univ. Calif. Riverside Ent. Res. Museum UCRC ENT 142420”. The holotype is in good condition, complete, dissected under 3 coverslips.

Paratypes: USA, Michigan: Clinton Co. (same data as the holotype), 2 ♀ on points [MSUC, UCRC] and 1 ♀, 1 ♂ on slides [UCRC]. Ingham Co., Michigan State University Tree Research Center, 42°40'12"N, 84°28'12"W, 267 m, 14.viii.2014, T. R. Petrice, emerged in laboratory (East Lansing) from parasitized *Agrilus egenus* Gory eggs on black locust, *Robinia pseudoacacia*, twigs: emerged 22.viii.2014 [3 ♀ on points, MSUC, UCRC, USNM]; emerged 29.viii.2014 [3 ♀ on points, MSUC, UCRC, USNM, and 1 ♂ on slide, UCRC]; emerged 6.ix.2014 [1 ♀ on point, UCRC]; emerged 17.ix.2014 [1 ♂ on slide, UCRC].

##### Description.

FEMALE (holotype). Body dark brown to black except scutellum and propodeum brown; scape and pedicel brown, flagellum light brown; legs whitish or pale yellowish with wide brown bands on coxae, femora, and tibiae.

Frontovertex and mesonotum with faint mesh-like or lineolate sculpture [very difficult to see in dry-mounted specimens, best observed in slide-mounted ones (as in Fig. 25)]. Pronotum, mesoscutum, axillae, and scutellum with short, dusky setae; scutellum also with a pair of long, fine setae near posterior margin.

Head (as in Fig. 15, collapsed when air-dried) with ocelli in an obtuse triangle, posterior ocellus a little less than its diameter away from eye margin. Transfacial and inner orbital sutures present. Mandible 3-dentate, the inner tooth with two denticles; maxillary palpus 4-segmented, labial palpus 1-segmented (i.e., palpal formula 4–1).

Antenna (Fig. 12) inserted below lower eye margin. Radicle about 0.3× total scape length, rest of scape slender, 4.5× as long as wide, a little wider in the middle, with faint longitudinal sculpture. Pedicel longer than any funicle segment; F1–F5 slightly transverse, F1–F4 subequal in length, F5 a little longer and slightly wider than long; F6 the longest funicle segment, longer than wide; F1–F5 without mps, and F6 with 2 mps. Clava 3-segmented, about 2.3× as long as wide and almost as long as funicle; first claval segment with 1 mps, second and third segments each with 3 mps.

Mesosoma a little shorter than gaster (Fig. 13). Mesoscutum about 1.7× as wide as long. Scutellum a little wider than long, a little shorter than mesoscutum; scutellar placoid sensilla closer to the posterior margin of scutellum and close to each other.

Wings (Fig. 31) not abbreviated, forewing extending far beyond apex of gaster. Forewing 2.1× as long as wide, hyaline; marginal setae very short; disc densely setose, linea calva interrupted posteriorly by an irregular row of setae, filum spinosum present. Hindwing 4.2× as long as wide, hyaline; longest marginal seta 0.3× maximum wing width.

Mesotibial spur a little longer than mesobasitarsus.

Ovipositor occupying a little more than 0.5× length of gaster, exserted markedly beyond gastral apex (by 0.2× own length) (Fig. 13); ovipositor length:metatibia length ratio 1.2:1. Outer plate of ovipositor with two subapical setae.

Measurements of the holotype (mm, as length or length:width). Body (of the dry-mounted specimen prior to slide-mounting): 0.462; mesosoma: 0.233; gaster: 0.245; ovipositor: 0.173. Antenna: radicle: 0.03; rest of scape: 0.103; pedicel: 0.045; F1: 0.012; F2: 0.012; F3: 0.011 (0.012); F4: 0.012; F5: 0.015; F6: 0.03; clava: 0.103. Forewing: 0.495:0.234; longest marginal seta: 0.021; hindwing: 0.357:0.085; longest marginal seta: 0.025.

**Figures 33–35.**
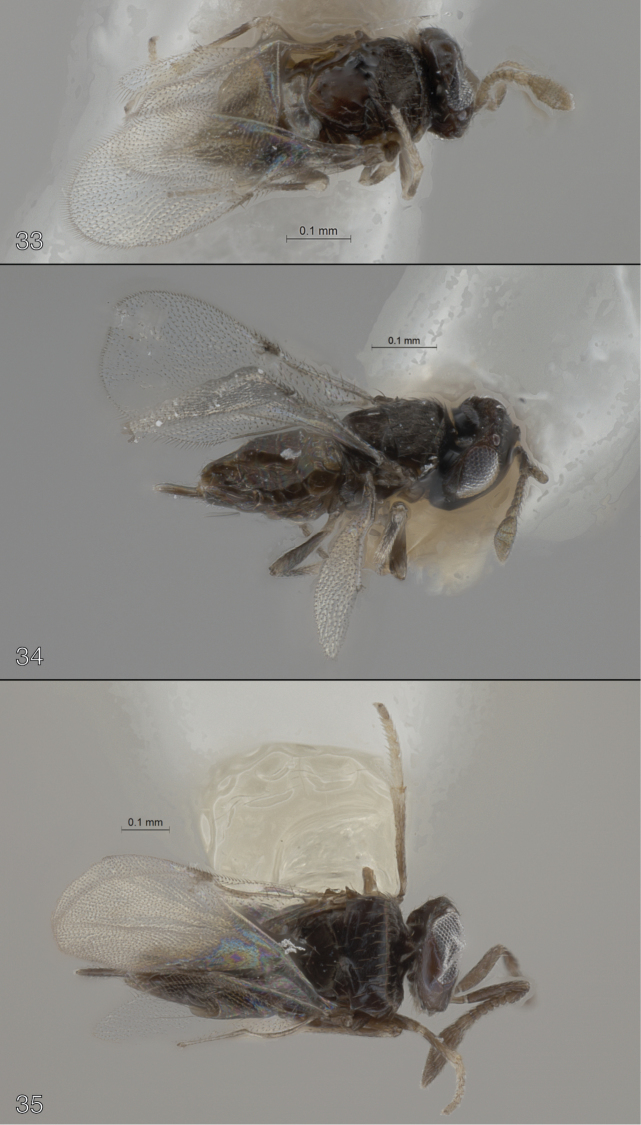
**33–34** *Oobius minusculus* (paratype females) **33** dorsal habitus (from *Agrilus subcinctus*) **34** lateral habitus (from *Agrilus egenus*) **35** *Oobius whiteorum* (paratype female), dorsal habitus.

Variation (paratypes). Body length 0.43–0.46 mm (dry-mounted specimens from *Agrilus subcinctus*, Fig. 33) or 0.46–0.53 mm (critical-point dried specimens from *Agrilus egenus*, Fig. 34). In the latter specimens, legs (except tarsi) are somewhat darker (mostly brown), scape (minus radicle) of the female antenna is about 5.0× as long as wide, and clava is about 2.5× as long as wide. Mandibles are identical for specimens reared from both host species, and there is no doubt that they are conspecific. In all specimens, F6 is sometimes slightly paler than other flagellomeres but not contrastingly, still almost concolorous or often concolorous.

MALE (paratype from *Agrilus subcinctus*). Head dark brown, mesosoma and gaster dark brown to black except mesoscutum with a brownish tinge, base of gaster whitish; antenna with scape and pedicel brown to dark brown, flagellum light brown. Antenna (Fig. 16) with scape minus radicle 2.9× as long as wide; F2–F4 more or less subequal in length, F1 and F5 slightly longer, F6 the longest funicle segment; F2–F4 without mps, F1, F5, F6 and clava with mps; flagellar segments with very long setae (slightly longer than each funicle segment’s width); clava entire, 2.6× as long as wide, a little wider than funicle segments. Mesosoma (Fig. 25) about as long as gaster. Forewing 2.0× as long as wide, hyaline. Genitalia (Fig. 14) typical for the genus.

Variation (paratypes from *Agrilus egenus*). Body length 0.4–0.5 mm (critical-point dried specimens).

##### Diagnosis.

This species is similar to the European *Oobius zahaikevitshi* Trjapitzin (Figs 19, 30), whose type locality is Zhuravlivka, Vinnytsia Oblast, Ukraine, where it was reared from eggs of *Agrilus viridis* (Linnaeus) on *Carpinus betulus* (Trjapitzin 1963). *Oobius zahaikevitshi* was recently well illustrated by Gumovsky et al. (2013). It was recorded from several European countries and *Agrilus* spp. hosts, listed by Trjapitzin and Volkovitsh (2011) and Noyes (2014). However, we are not absolutely confident that all these records are correct: it is quite possible that they might represent a complex of more than one cryptic species that are difficult to distinguish without supporting molecular data and thorough morphological studies based on good quality slide-mounted specimens. Proportions of funicle segments of the female antenna seem to be somewhat different between the specimens of *Oobius zahaikevitshi* from Ukraine illustrated by Trjapitzin (1963) and Gumovsky et al. (2013), in which F5 is about as long as wide, and the examined specimens from Volgograd Province of Russia, in which F5 is a little wider than long (Fig. 30).

*Oobius minusculus* differs from *Oobius zahaikevitshi* in having the palpal formula 4–1, a relatively smaller F5 of the female antenna and also by F6 being longer than wide and almost concolorous or often concolorous with other flagellomeres (Fig. 12). In contrast, the palpal formula for *Oobius zahaikevitshi* is 3–1, F5 is relatively larger, and F6 is about as long as wide and contrastingly lighter than other flagellomeres (Fig. 30), as also described and illustrated in Trjapitzin (1963) and Gumovsky et al. (2013).

*Oobius minusculus* is the only described native Nearctic species of *Oobius* s. str., as characterised by Noyes (2010) in having the outer plate of the ovipositor being relatively short and apically rounded with paired subapical setae (one long and one short), in which this new taxon fits well. In the key by Trjapitzin and Volkovitsh (2011) to the world species of *Oobius* (s. str.), it keys to *Oobius zahaikevitshi*. In Noyes (2010), *Oobius minusculus* tentatively keys (although it really does not key to any of the included Neotropical species) to the same couplet with *Oobius xochipili* Noyes and *Oobius zagan* Noyes from Costa Rica, from both of which it differs by F5 of the female antenna being much less transverse, just slightly wider than long (Fig. 12) whereas in *Oobius xochipili* and *Oobius zagan* F5 is anelliform, much wider than long (Noyes 2010).

##### Etymology.

The name of this new taxon is an adjective referring to its small size.

##### Hosts.

*Agrilus subcinctus* on ash (*Fraxinus* spp.) and *Agrilus egenus* on black locust (*Robinia pseudoacacia*).

##### Notes on biology.

Originally reported by Petrice et al. (2009) as *Avetianella* sp. that parasitized *Agrilus subcinctus* eggs. The second author has never found this parasitoid to overwinter in *Agrilus subcinctus* eggs. However, collections of *Agrilus egenus* eggs found overwintering *Oobius minusculus* larvae in eggs. This species likely attacks other *Agrilus* spp. in North America, and has multiple generations per year.

##### Comments.

The following specimens of *Oobius zahaikevitshi* were examined: Bulgaria, Plovdiv Prov., Klisura, 27.vi–8.vii.1975, A. Atanasov, from eggs of *Agrilus cuprescens* (Ménétriés) on *Rosa* sp. [1 ♂, BMNH] (det. V. A. Trjapitzin 1977). Russia, Volgograd Prov. (oblast’), Krasnoarmeyskiy District (rayon), environs of Volgograd, vi.1971, A. M. Makhmadziyoev (Makhmadzieev), from eggs of *Agrilus viridis* on *Acer tataricum* [1 ♀, BMNH; 5 ♀, UCRC] (det. V. A. Trjapitzin 1977 and 1975, respectively).

#### Oobius nearcticus

Taxon classificationAnimaliaHymenopteraEncyrtidae

(Trjapitzin, 1977)

Figures 9–10 , 24

Szelenyiola nearctica Trjapitzin 1971: 160–161. Type locality: Blodgett Forest (8 mi. E. of Georgetown), University of California Blodgett Forest Research Station, El Dorado Co., California, USA.
Oobius nearcticus (Trjapitzin): Noyes 2010: 662, 668, 671.

##### Type material examined.

Holotype female [EMEC] on point mount with following four labels: “UC Blodgett Forest 8 mi E. Georgetown, El Dorado Co., California Coll. F. M. Stephen 1970”, “Traps A-1094”, [red] “Holotypus *Szelenyiola nearctica* Trjapitzin”, “U.C. Berkeley EMEC 82,322”. Paratype female [EMEC] on point with following five labels: “UC Blodgett Forest 8 mi E. Georgetown, El Dorado Co., California Coll. F. M. Stephen 1970 A-1094 Traps”, “Head with appendages on slide No. 1955”, “Also forewing”, “Paratypus”, [red] “*Szelenyiola nearctica* Trjapitzin ♀”.

##### Distribution.

USA (California) (Trjapitzin 1977).

##### Hosts.

Unknown.

##### Comments.

The holotype (Fig. 24) is missing its left hindwing and the apical 2/3 of the left forewing.

#### Oobius whiteorum

Taxon classificationAnimaliaHymenopteraEncyrtidae

Triapitsyn sp. n.

http://zoobank.org/00F395AF-FD46-4102-A70E-BBD69B5176C1

Figures 11b , 26–29 , 32 , 35

Avetianella sp.: Loerch and Cameron 1983: 1798–1799 (egg parasitoid, host information); Trjapitzin 2001: 738 (list).

##### Type material.

Holotype female [UCRC] on slide (Fig. 11b) with following five labels: “USA, Pennsylvania, Venango Co., Bullion, 8.VII.1982, C. R. Loerch, Ex. *Agrilus anxius* Gory eggs”, “Mounted by V. V. Berezovskiy 2014 in Canada balsam”, “*Avetianella* sp. (Encyrtidae) Det. J. LaSalle”, [magenta] “*Oobius whiteorum* Triapitsyn HOLOTYPE ♀”, [database label] “Univ. Calif. Riverside Ent. Res. Museum UCRC ENT 401252”. The holotype is in good condition, complete, dissected under 3 coverslips.

Paratypes: same data as the holotype, 4 ♀ on points and 1 ♂ on slide [UCRC].

##### Description.

FEMALE (holotype). Body somewhat flattened, dark brown to black; appendages brown except tarsi light brown; scape and pedicel a little darker than flagellum, and F6 just slightly lighter than other flagellar segments but still brown.

Frontovertex and mesonotum with faint mesh-like sculpture [very difficult to see in dry-mounted specimens]. Pronotum, mesoscutum, axillae, and scutellum with short, dusky setae; scutellum also with a pair of long, fine setae near posterior margin.

Head (Fig. 28) with ocelli in an obtuse triangle, posterior ocellus about its diameter away from eye margin. Transfacial and inner orbital sutures absent. Mandible 3-dentate; palpal formula 4–3.

Antenna (Fig. 26) inserted below lower eye margin. Radicle about 0.2× total scape length, rest of scape slender, 4.1–4.2× as long as wide, a little wider in the middle, with faint longitudinal sculpture. Pedicel longer than any funicle segment. F1–F3 about as long as wide, F4–F6 longer than wide; F1–F3 subequal, F4–F6 each progressively a little longer than the preceding funicle segment; F1–F3 without mps, F4 with 1 mps, F5 with 2 mps, and F6 with 3 or 4 mps. Clava 2.8× as long as wide, and slightly shorter than combined length of F2–F6; each claval segment with 3 mps; apical claval segment obliquely truncate ventrally.

Mesosoma (Fig. 28) shorter than gaster. Mesoscutum about 1.6× as wide as long. Scutellum wider than long, almost as long as mesoscutum.

Wings (Fig. 27) not abbreviated, forewing extending far beyond apex of gaster. Forewing 2.1× as long as wide, hyaline; marginal setae very short; disc densely setose, linea calva interrupted posteriorly by rows of setae, filum spinosum present. Hindwing 3.7–3.8× as long as wide, hyaline; longest marginal seta 0.18× maximum wing width.

Mesotibial spur as long as mesobasitarsus.

Ovipositor occupying about 0.5× length of gaster, exserted markedly beyond gastral apex (by 0.36× total ovipositor length); ovipositor length:metatibia length ratio 1.3:1. Outer plate of ovipositor with 1 subapical seta.

Measurements of the holotype (mm, as length or length:width). Body (of the dry-mounted specimen prior to slide-mounting): 0.66; head: 0.19; mesosoma: 0.313; gaster: 0.35; ovipositor: 0.283. Antenna: radicle: 0.039; rest of scape: 0.151; pedicel: 0.06; F1: 0.021; F2: 0.021; F3: 0.021; F4: 0.028; F5: 0.035; F6: 0.044; clava: 0.155. Forewing: 0.677:0.314; longest marginal seta: 0.021; hindwing: 0.5:0.133; longest marginal seta: 0.024.

Variation (paratypes). Body (Fig. 35) length 0.66–0.75 mm (dry-mounted specimens).

MALE (paratype). Body length (of the dry-mounted specimen prior to slide-mounting) 0.66 mm. Head and mesosoma dark brown, gaster brown; scape and pedicel brown, flagellum light brown; legs light brown to brown. Antenna (Fig. 32) with scape minus radicle 3.2× as long as wide; funicle segments longer than wide, more or less subequal in length (F5 and particularly F6 slightly longer), F1 and F2 without mps, F3 with or without mps, F4–F6 and clava with mps; flagellar segments with very long setae (slightly longer than each funicle segment’s width and about as long as width of clava); clava entire, 2.6–2.7× as long as wide, a little wider than funicle segments. Mesosoma about as long as gaster. Forewing 1.9× as long as wide, hyaline. Hindwing 3.5× as long as wide, hyaline. Genitalia (Fig. 29) typical for the genus.

##### Diagnosis.

Among the Nearctic species of *Oobius*, *Oobius whiteorum* is most similar to *Oobius dahlsteni*, from which it differs by the proportions of the funicle segments of the female antenna, as indicated in the key. In Trjapitzin’s (2001) key to the world species of the former genus *Avetianella* (s. str.) in which this new species mostly fits, as characterised by Noyes (2010) in having the outer plate of the ovipositor being conspicuously distally elongate and ribbon-like and always with only a single subapical seta, it keys to *Oobius dahlsteni*. *Oobius whiteorum* differs from *Oobius depressus*, to which it is also somewhat similar, by a relatively less flattened body and by the much smaller body size in females; according to Girault (1916), the body length of the latter species is 1.15 mm. *Oobius whiteorum* differs from the North American species, but Neotropical species *Oobius hasmik* (Trjapitzin), known from Mexico (Trjapitzin 2001) and also Costa Rica (Noyes 2010), by the “closed” linea calva (Fig. 27) on the forewing (“open”, not interrupted, in *Oobius hasmik*, Fig. 21) and also by the different proportions of the scape of the female antenna (Figs 26 and 20, respectively). In Noyes (2010), *Oobius whiteorum* keys to the same couplet with *Oobius lutron* Noyes from Costa Rica and Brazil, from which it differs by each of F4–F6 of the female antenna being of different length and longer than wide (Fig. 26) whereas in *Oobius lutron* F4–F6 are subequal and each quadrate or hardly longer than broad (Noyes 2010).

##### Host.

*Agrilus anxius* Gory on European white birch (*Betula pendula*).

##### Etymology.

This species is named in honor of Lisa and Michael White of Chicago, Illinois, USA, good friends of the author’s family.

##### Comments.

According to Loerch and Cameron (1983), additional voucher specimens of the egg parasitoids of *Agrilus anxius* were deposited by them in PSUC; any of them belonging to this species are non-type specimens. Unfortunately, due to a renovation of the museum, point-mounted specimens in that collection are now inaccessible (A. Deans, personal communication).

The following paratypes [UCRC] of *Oobius hasmik* were examined, all collected at Las Barracas (~30 km E of Santiago, 23°28'02"N, 109°27'01"W, 50 m), Baja California Sur, Mexico: 1 ♀ on point with following five labels: “Mex. Baja Cal. Sur Las Barracas 17 - V - 1985”, “Coll. P. DeBach Pan trap”, “*Avetianella* ♀ Det. V. Trjapitzin May 1997”, [red] “Paratypus ♀ *Avetianella hasmik* Trjapitzin”, “Praep. micr. 22M” (an antenna, head, and a forewing were detached from this specimen; they are mounted on a slide with following two labels: “*Avetianella hasmik* ♀ Trjapitzin México: Baja California Sur, Las Barracas. Pan trap 17.V.1985 (Coll. P. DeBach) 22M Antena, cabeza, ala anterior”, [red] “Paratypus Avetianella ♀ hasmik Trjapitzin”); also 16 ♀, 1 ♂ on points, all collected by P. DeBach during 1985 and 1986, as indicated by Trjapitzin (2001).

#### Oobius sp.

Taxon classificationAnimaliaHymenopteraEncyrtidae

Oobius sp. (Not included in the key)
Oobius sp. n.: Trjapitzin et al. 2008: 186 (record from Mexico).
Oobius sp. n. aff. rudnevi (S. Nowicki, 1928): Trjapitzin and Volkovitsh 2011: 674 (list, Mexico).

##### Comments.

One female [UANL] of this undescribed species from Mexico, which has no host information, was mentioned by Trjapitzin and Volkovitsh (2011); however, they did not indicate the collecting locality so it is unknown from which part of that country it was found (Nearctic or Neotropical).

## Supplementary Material

XML Treatment for Oobius

XML Treatment for Oobius agrili

XML Treatment for Oobius buprestidis

XML Treatment for Oobius dahlsteni

XML Treatment for Oobius depressus

XML Treatment for Oobius longoi

XML Treatment for Oobius minusculus

XML Treatment for Oobius nearcticus

XML Treatment for Oobius whiteorum

XML Treatment for Oobius sp.

## References

[B1] Abell KJ Bauer LS Duan JJ Van Driesche R (2014) Long-term monitoring of the introduced emerald ash borer (Coleoptera: Buprestidae) egg parasitoid, *Oobius agrili* (Hymenoptera: Encyrtidae), in Michigan, USA and evaluation of a newly developed monitoring technique. Biological Control 79(1): 36–42. doi: 10.1016/j.biocontrol.2014.08.002

[B2] Annecke DP (1967) Three new southern African species of *Oobius* Trjapitzin, 1963 (Hymenoptera: Encyrtidae). Journal of Natural History 1(3): 319–325. doi: 10.1080/00222936700770291

[B3] Bauer LS Duan JJ Gould J (2014) XVII Emerald ash borer (*Agrilus planipennis* Fairmaire) (Coleoptera: Buprestidae). In: Van Driesche R Reardon R (Eds) The use of classical biological control to preserve forests in North America. United States Department of Agriculture, Forest Service, FHTET-2013-02, Morgantown, West Virginia, 189–209 http://www.fs.fed.us/foresthealth/technology/pdfs/FHTET-2013-2.pdf

[B4] Bauer LS Duan JJ Gould JG Van Driesche RG (in press) Progress in the classical biological control of *Agrilus planipennis* Fairmaire (Coleoptera: Buprestidae) in North America. The Canadian Entomologist.

[B5] Bray AM Bauer LS Poland TM Haack RA Cognato AI Smith JJ (2011) Genetic analysis of emerald ash borer (*Agrilus planipennis* Fairmaire) populations in Asia and North America. Biological Invasions 13: 2869–2887. doi: 10.1007/s10530-011-9970-5

[B6] Gibson GAP (1997) Chapter 2. Morphology and terminology. In: Gibson GAP Huber JT Woolley JB (Eds) Annotated keys to the genera of Nearctic Chalcidoidea (Hymenoptera). NRC Research Press, Ottawa, Ontario, Canada, 16–44.

[B7] Girault AA (1916) Descriptions of and observations on some chalcidoid Hymenoptera – II. The Canadian Entomologist 48(10): 337–344. doi: 10.4039/Ent48337-10

[B8] Gordh G Trjapitzin VA (1981) Taxonomic studies of the Encyrtidae with descriptions of new species and a new genus (Hymenoptera, Chalcidoidea). University of California Publications in Entomology 93: 1–64.

[B9] Gumovsky AV Simutnik SA Prokhorov AV (2013) Life-history review of *Oobius zahaikevitshi* Trjapitzin, 1963 (Hymenoptera: Encyrtidae), an egg parasitoid of jewel beetles (Coleoptera: Buprestidae). Russian Entomological Journal 22(3): 181–188.

[B10] Haack RA Jendek E Liu HP Marchant K Petrice T Poland TM Ye H (2002) The emerald ash borer: a new exotic pest in North America. Newsletter of the Michigan Entomological Society 47(3–4): 1–5.

[B11] Hanks LM Gould JR Paine TD Millar JG Wang Q (1995) Biology and host relations of *Avetianella longoi* (Hymenoptera: Encyrtidae), an egg parasitoid of the eucalyptus longhorned borer (Coleoptera: Cerambycidae). Annals of the Entomological Society of America 88(5): 666–671. doi: 10.1093/aesa/88.5.666

[B12] Liao DX Li XL Pang XF Chen TL (1987) Hymenoptera: Chalcidoidea (1). Economic Insect Fauna of China No 34: x+241 pp.

[B13] Liu HP Bauer LS Miller DL Zhao TH Gao RT Song L Luan Q Jin R Gao C (2007) Seasonal abundance of *Agrilus planipennis* (Coleoptera: Buprestidae) and its natural enemies *Oobius agrili* (Hymenoptera: Encyrtidae) and *Tetrastichus planipennisi* (Hymenoptera: Eulophidae) in China. Biological Control 42: 61–71. doi: 10.1016/j.biocontrol.2007.03.011

[B14] Loerch CR Cameron EA (1983) Natural enemies of immature stages of the bronze birch borer, *Agrilus anxius* (Coleoptera: Buprestidae), in Pennsylvania. Environmental Entomology 12: 1798–1801. doi: 10.1093/ee/12.6.1798

[B15] Luhring KA Paine TD Millar JG Hanks LM (2000) Suitability of the eggs of two species of eucalyptus longhorned borers (*Phoracantha recurva* and *P. semipunctata*) as hosts for the parasitoid *Avetianella longoi* Siscaro. Biological Control 19: 95–104. doi: 10.1006/bcon.2000.0853

[B16] MacRae TC (1991) The Buprestidae (Coleoptera) of Missouri. Insecta Mundi 5: 101–126.

[B17] Mapbiocontrol (2014) Emerald ash borer biocontrol release and recovery. http://www.mapbiocontrol.org/

[B18] Nelson GH Walters GC Haines RD Bellamy CL (2008) A catalog and bibliography of the Buprestoidea of America North of Mexico. The Coleopterists Society Special Publ. No. 4: 1–274.

[B19] Noyes JS (2010) Encyrtidae of Costa Rica (Hymenoptera: Chalcidoidea), 3. Subfamily Encyrtinae: Encyrtini, Echthroplexiellini, Discodini, Oobiini and Ixodiphagini, parasitoids associated with bugs (Hemiptera), insect eggs (Hemiptera, Lepidoptera, Coleoptera, Neuroptera) and ticks (Acari). Memoirs of the American Entomological Institute 84: 1–848.

[B20] Noyes JS (2014) Universal Chalcidoidea database. WWW publication The Natural History Museum, London http://www.nhm.ac.uk/research-curation/projects/chalcidoids/index.html [accessed 12.i.2015]

[B21] Petrice TR Haack RA Strazanac JA Lelito JP (2009) Biology and larval morphology of *Agrilus subcinctus* (Coleoptera: Buprestidae), with comparisons to the emerald ash borer, *Agrilus planipennis*. The Great Lakes Entomologist 42: 173–184.

[B22] Siscaro G (1992) *Avetianella longoi* sp. n. (Hymenoptera Encyrtidae) egg parasitoid of *Phoracantha semipunctata* F. (Coleoptera Cerambycidae). Bollettino di Zoologia Agraria e Bachicoltura (2) 24(2): 205–212.

[B23] Trjapitzin VA (1963) Species of the genus *Oobius* gen. n. (Hymenoptera, Encyrtidae) in the USSR. Acta Entomologica Musei Nationalis Pragae [Sborník Entomologického Oddeleni Národního Musea v Praze] 35: 543–547.

[B24] Trjapitzin VA (1968) [A survey of the encyrtid fauna (Hym. Encyrtidae) of the Caucasus]. Trudy Vsesoyuznogo Entomologicheskogo Obshchestva 52: 43–125 [In Russian]

[B25] Trjapitzin VA (1971) [A Nearctic representative of the genus *Avetianella* Trjapitzin, 1968 (Hymenoptera, Encyrtidae)]. Entomologicheskoe Obozrenie 50(4): 890–892 [In Russian]

[B26] Trjapitzin VA (1977) New genera and species of parasitic Hymenoptera of the family Encyrtidae (Hymenoptera: Chalcidoidea). Folia Entomologica Hungarica 30(1): 153–166.

[B27] Trjapitzin VA (2001) [A review of encyrtids of the genus *Avetianella* Trjapitzin, 1968 (Hymenoptera, Encyrtidae) of the world fauna with description of a new species from Mexico]. Entomologicheskoe Obozrenie 80(3): 734–739 [In Russian]

[B28] Trjapitzin VA Gordh G (1984) [Taxonomic notes on the Neotropic genus *Habrolepoidea* (Hymenoptera, Encyrtidae) and on species erroneously referred to it]. Zoologicheskiy Zhurnal 63(8): 1273–1277 [In Russian]

[B29] Trjapitzin VA Myartseva SN Ruíz-Cancino E Coronado-Blanco JM (2008) Clave de géneros de Encyrtidae (Hymenoptera: Chalcodoidea) de México y un catálogo de las especies. Serie Avispas parasíticas de plagas y otros insectos. Universidad Autónoma de Tamaulipas, Ciudad Victoria, Tamaulipas, México, 265 pp.

[B30] Trjapitzin VA Volkovitsh MG (2011) A review of species of the genus *Oobius* Trjapitzin, 1963 (Hymenoptera, Encyrtidae) egg parasitoids of jewel beetles, longicorn beetles (Coleoptera, Buprestidae, Cerambycidae), and robber flies (Diptera, Asilidae). Entomological Review 91(5): 670–676 [English translation, originally published in Russian in Entomologicheskoe Obozrenie 90 (1): 226–234]

[B31] Zhang Y Huang D (2004) A review and an illustrated key to genera of Encyrtidae (Hymenoptera: Chalcidoidea) from China. Science Press, Beijing, China, 166 pp.

[B32] Zhang Y-Z Huang D-W Zhao T-H Liu H-P Bauer LS (2005) Two new species of egg parasitoids (Hymenoptera: Encyrtidae) of wood-boring beetle pests from China. Phytoparasitica 33(3): 253–260. doi: 10.1007/BF02979863

